# Frequency of *CDH1 *germline mutations in gastric carcinoma coming from high- and low-risk areas: metanalysis and systematic review of the literature

**DOI:** 10.1186/1471-2407-12-8

**Published:** 2012-01-06

**Authors:** Giovanni Corso, Daniele Marrelli, Valeria Pascale, Carla Vindigni, Franco Roviello

**Affiliations:** 1 Department of Human Pathology and Oncology, section of General Surgery and Surgical Oncology, Translational Research Laboratory, University of Siena, Viale Bracci, 53100 Siena, Italy; 2 Unit of Pathology, Hospital Santa Maria alle Scotte, Azienda Ospedaliera Universitaria Senese, Siena, Italy

**Keywords:** Gastric cancer, E-cadherin, Germline mutation, Risk area

## Abstract

**Background:**

The frequency of E-cadherin germline mutations in countries with different incidence rates for gastric carcinoma has not been well established. The goal of this study was to assess the worldwide frequency of *CDH1 *germline mutations in gastric cancers coming from low- and high-risk areas.

**Methods:**

English articles using MEDLINE access (from 1998 to 2011). Search terms included *CDH1*, E-cadherin, germline mutation, gastric cancer, hereditary, familial and diffuse histotype.

The study included all E-cadherin germline mutations identified in gastric cancer patients; somatic mutations and germline mutations reported in other tumors were excluded.

The method of this study was scheduled in accordance with the "PRISMA statement for reporting systematic reviews and meta-analyses". Countries were classified as low- or middle/high risk-areas for gastric carcinoma incidence. Statistical analysis was performed to correlate the *CDH1 *mutation frequency with gastric cancer incidence areas.

**Results:**

A total of 122 E-cadherin germline mutations have been identified; the majority (87.5%) occurred in gastric cancers coming from low-risk areas. In high-risk areas, we identified 16 mutations in which missense mutations were predominant. (68.8%). We verified a significant association between the mutation frequency and the gastric cancer risk area (*p *< 0.001: overall identified mutations in low- vs. middle/high-risk areas).

**Conclusions:**

E-cadherin genetic screenings performed in low-risk areas for gastric cancer identified a higher frequency of *CDH1 *germline mutations. This data could open new approaches in the gastric cancer prevention test; before proposing a proband candidate for the *CDH1 *genetic screening, geographic variability, alongside the family history should be considered.

## Background

*CDH1 *germline mutations are associated with the development of the autosomal cancer syndrome namely Hereditary Diffuse Gastric Cancer (HDGC) [1,2]; about 25-30% of families fulfilling the clinical criteria for HDGC established by the International Gastric Cancer Linkage Consortium (IGCLC) have constitutional alterations of the *CDH1 *gene [3]. *CDH1 *germline mutations can also be identified in sporadic early onset GC (EOGC) in less than 4% of patients 35 years of age at the time of diagnosis, presenting as *de novo *mutations [4].

Different patterns of *CDH1 *germline mutations have been described as truncating, deletion, insertion, splice site, non sense, silence, and at last, missense alterations [5].; for *CDH1 *non-missense mutations, the penetrance rate is high, with an estimated risk of > 80% [3]., in missense mutation carriers, the estimated cancer-risk is unknown. *CDH1 *missense mutations are rather frequently detected in HDGC and in EOGC, and they can be classified as neutral or pathogenic variants [6].

The worldwide incidence of gastric carcinoma is extremely heterogeneous and the causes of these differences are still unclear; it has been reported that GC presents different characteristics considering tumors coming from low- and high-risk area [7]. In GC patients coming from high-risk areas environmental factors, as specific foods, are more probably associated with the gastric carcinogenesis [8,9] in which genetic factors, as *CDH1 *or *TP53 *germline mutations, are very rarely identified [10-15]. To assess the *CDH1 *germline mutation frequency in low- and high-risk areas for GC, we reviewed all E-cadherin constitutional alterations identified from 1998 to date, considering the worldwide geographic distribution. We focused on low- and high-risk areas of gastric carcinoma in particular.

## Methods

### Study accuracy and approach

The accuracy of this systematic review and meta-analysis was assessed using the checklist of items in accordance with the "Preferred Reporting Items for Systematic reviews and Meta-Analyses (PRISMA) statement" [16].

**Figure 1:**
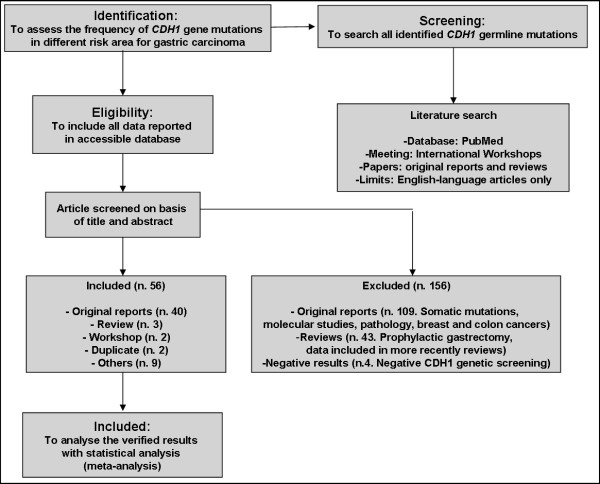
**Flow diagram of study selection and information through different phases of the systematic review and meta-analysis**. (The diagram was structured in accord with the Digestive Disease Week; United European Gastroenterology Week and with the PRISMA statement)

In particular, we scheduled this systematic review and meta-analysis as follows: a) Identification: to assess the *CDH1 *germline mutation frequency in different risk areas for GC; b) Screening: to search all identified *CDH1 *germline mutations, as well as in patients with DGC as in asymptomatic carriers; c) Eligibility: to include all data reported in an accessible source bank (the National Library of Medicine's MEDLINE database); d) Included: to analyse the obtained results with statistical analysis (Figure 1).

### Study population

In this study we considered all countries in which an independent *CDH1 *genetic screening has been performed, aiming for germline mutation search.

We subdivided the world areas in two groups: low (group A) and middle-high incidence (group B) areas, considering the GC incidence data reported by Ferlay J. and Colleagues [17]. Among group A, we identified these countries: USA, Spain, France, Ireland, Canada, United Kingdom, New Zealand, Germany, Finland, Pakistan, Sweden, the Netherlands, Austria and Iran. This group also included some cases defined only as "European" and without specific ancestry. We excluded that this part is comes from the European area with middle-high GC risk (as North of Portugal, Central Italy, and Eastern Europe). Group B includes these zones: Italy (Tuscany), Portugal (North), Japan, China, Korea, South America, Lithuania and Poland.

### CDH1 germline mutation search and selected articles

We revised all *CDH1 *germline mutations reported in MEDLINE from March 1998 to November 2010, through analysis of original reports and reviews of the literature edited in the English language. Moreover, we also included data from two international workshops of the IGCLC (in 1999 and in 2010) [2,3]. Whenever sufficient, we also considered information obtained from abstract in the English language with the main text edited in other languages; a total of 156 papers were selected for this study. The reported *CDH1 *constitutional alterations are defined as truncating, deletion, insertion, splice site, non-sense, and missense mutations (Table 1); we grouped these mutations as *non-missense *(truncating, deletion, insertion, splice site and non sense) or *missense *mutations. This study excluded all *CDH1 *somatic mutations identified in gastric carcinoma, and the germline alterations found in primary lobular breast carcinoma as well as in mucinous colon cancer. We considered also mutations namely in review papers as "unpublished results" [5,18].

**Table 1:** *CDH1 *germline mutations detected to date

Reference	*CDH1 *mutation	Exon/Intron	Mutation Type	Ancestry
[19]	5'UTR(-117)G > A	Promotor	5'UTR substitution	USA (1)
[19]	5'UTR(-71)C > G	Promotor	5'UTR substitution	USA (1)
[4]	-63 C > A	Promotor	5'UTR substitution	Italy (1)
[20]	Del 5'UTR-ex 1	Promotor	Deletion	Spain (1)
[20]	del ex 1-2	Exon 1-2	Deletion	Ireland (1), Canada (1) Lithuania (1)
[5]	2 T > C	Exon 1	Missense	Unknow (1)
[21]	3 G > C	Exon 1	Nonsense	Canada (1)
[19]	41delT	Exon 1	Deletion	USA (1)
[22]	45insT	Exon 1	Insertion	UK (1)
[5]	46insTGC	Exon 1	Insertion	Unknow (1)
[19]	48 + 5 G > C	Intron 1	Splice Site	USA (1)
[19]	48 + 15 C > G	Intron 1	Splice Site	USA (1)
[18,23,24]	49-2A > G	Intron 1	Splice Site	Europe (1), UK (1), Ireland (1)
[25]	53delC	Exon 2	Deletion	Europe (1)
[23]	59 G > A	Exon 2	Non sense	UK (1)
[26]	70 G > T	Exon 2	Non sense	USA (1)
[27]	185 G > T	Exon 3	Missense	Japan (1)
[28]	187 C > T	Exon 3	Non sense	USA (1)
[1]	190 C > T	Exon 3	Non sense	New Zealand (1)
[29]	283 C > T	Exon 3	Non sense	France (1)
[18]	353 C > G	Exon 3	Missense	Europe (1)
[30]	372delC	Exon 3	Deletion	Germany (1)
[31]	377delC	Exon 3	Deletion	Unknow (1)
[32]	382delC	Exon 3	Deletion	Europe (1)
[19]	388 + 26 C > T	Intron 3	Splice Site	USA (1)
[31]	515 C > G	Exon 4	Missense	Unknown (1)
[33]	525 C > G	Exon 4	Missense	Finland (1)
[6]	532-18 C > T	Intron 4	Splice site	UK (1), Portugal (1)
[26]	586 G > T	Exon 5	Non sense	Europe (1)
[32]	531 + 1 G > A	Intron 5	Splice site	Europe (1)
[34]	531 + 2 T > A	Intron 5	Splice site	France (1)
[35]	641 T > C	Exon 5	Missense	Europe (1)
[4]	670 C > T	Exon 5	Missense	Italy (1)
[18]	715 G > A	Exon 6	Missense	Europe (1)
[36]	731A > G	Exon 6	Missense	Korea (1)
[5]	753insG	Exon 6	Insertion	Unknown (1)
[31]	808 T > G	Exon 6	Missense	Unknown (1)
[22]	832 G > A	Exon 6	Splice site	Pakistan (1)
[37]	833-2A > G	Inton 6	Splice site	USA (1)
[32]	892 G > A	Exon 7	Missense	Europe (1)
[21,38]	1003 C > T	Exon 7	Non sense	Sweden (1), Canada (2)
[1]	1008 G > T	Exon 7	Splice site	New Zealand (1)
[22,39]	1018A > G	Exon 8	Missense	Europe (1), China (1)
[5]	1023 T > G	Exon 8	Nonsense	Unknown (1)
[21]	1063delT	Exon 8	Deletion	Canada (1)
[32]	1064insT	Exon 8	Insertion	UK (1)
[18]	1107delC	Exon 8	Deletion	Spain (1)
[40]	1118 C > T	Exon 8	Missense	Italy (1)
[32,41]	1134del8ins5	Exon 8	Deletion/Insertion	Netherlands (1)
[18,34]	1137 G > A	Exon 8	Splice site	Europe (2)
[26]	1137 + 1 G > A	Intron 8	Splice site	USA (1)
[32,42]	1212delC	Exon 9	Deletion	Europe (1), USA (1)
[32]	1226 T > C	Exon 9	Missense	Europe (1)
[43]	1243A > C	Exon 9	Missense	Japan (1)
[21]	1285 C > T	Exon 9	Missense	Canada (1)
[32]	1226 T > C	Exon 9	Missense	Europe (1)
[44]	1306_1303insA, 1306_1307delTT	Exon 9	Del/Ins	Austria (1)
[18]	1391-1392delTC	Exon 10	Deletion	Europe (1)
[36]	1460 T > C	Exon 10	Missense	Korea (1)
[45]	1466insC	Exon 10	Insertion	UK (1)
[22]	1472insA	Exon 10	Insertion	UK (1)
[32]	1476delAG	Exon 10	Deletion	Europe (1)
[26]	1487del7	Exon 10	Deletion	New Zealand (1)
[46]	1507 C > T	Exon 10	Non sense	China (1)
[25]	1565 + 1 G > T	Intron 10	Splice site	New Zealand (1)
[25]	1565 + 2insT	Intron 10	Insertion	USA (1)
[26]	1588insC	Exon 11	Insertion	USA (1)
[47]	1610delC	Exon 11	Deletion	Spain (1)
[48]	1619insG	Exon 11	Insertion	Germany (1)
[31]	1682insA	Exon 11	Insertion	Ireland (1)
[25]	1710delT	Exon 11	Deletion	USA (1)
[28]	1711insG	Exon 11	Insertion	USA (1)
[32]	1711 + 5 G > A	Intron 11	Splice site	Europe (1)
[49]	1774 G > A	Exon 12	Missense	Sweden (1)
[32]	1779insC	Exon 12	Insertion	Europe (1)
[21,25,28]	1792 C > T	Exon 12	Non sense	UK (1)
[31]	1795A > T	Exon 12	Missense	Unknown (1)
[32]	1779insC	Exon 12	Insertion	Europe (1)
[6]	1849 G > A	Exon 12	Missense	USA (2)
[31]	1876 T > A	Exon 12	Missense	Unknown (1)
[6,18,41]	1901 C > T	Exon 12	Missense/splice site	UK (1), New Zealand (1), Portugal (1)
[31]	1913 G > A	Exon 12	Nonsense	Spain (1)
[19]	1937-13 T > C	Intron 12	Splice Site	USA (1)
[32]	2061delTG	Exon 13	Deletion	Europe (1)
[37]	2064delTG	Exon 13	Deletion	USA (1)
[1,18]	2095 C > T	Exon 13	Non sense	New Zealand (1), China (1)
[21]	2161 C > G	Exon 13	Splice site	Canada (1)
[31]	2164 + 5 G > A	Exon 14	Splice site	Unknwon (1)
[20]	del 14-16	Ex 14-16	Deletion	Europe (1)
[32]	2195 G > A	Exon 14	Missense	Europe (1)
[31]	2245 C > T	Exon 14	Missense	Colombia (1)
[50]	2269 G > A	Exon 14	Missense	Portugal (1)
[51]	2275 G > T	Exon 14	Nonsense	Iran (1)
[21]	2276delG	Exon 14	Deletion	Canada (1)
[5]	2287 G > T	Exon 14	Nonsense	Unknown (1)
[25]	2295 + 5 G > A	Intron 14	Splice site	Europe (1)
[32]	2310delC	Exon 15	Deletion	Europe (1)
[31]	2343A > T	Exon 15	Missense	UK (1)
[1]	2381insC	Exon 15	Insertion	New Zealand (1)
[31]	2392 G > A	Exon 15	Missense	Unknown (1)
[37]	2395delC	Exon 15	Deletion	USA (1)
[48]	2396 C > G	Exon 15	Missense	Germany (1)
[31]	2398delC	Exon 15	Deletion	Ireland (2)
[19]	2439 + 31 G > A	Intron 15	Splice site	USA (1)
[52]	2399delG	Exon15	Deletion	USA (1)
[18]	2440-6 C > G	Intron 15	Splice site	Europe (1)
[53]	2494 G > A	Exon 16	Missense	Japan (1)
[20]	Del ex 16	Exon 16	Deletion	Europe (1)

We used the following search terms to search all reports, reviews and databases: E-cadherin; *CDH1 *gene; germline mutation, and genetic screening; HDGC, and IGCLC; familial GC; diffuse histotype; Maori kindred, prophylactic gastrectomy.

### Assessment of risk bias

We also evaluated the possibility that the available data is biased; in particular, we considered two kinds of possible bias: a) in case selection, specifically by the literature reviewer and b) from literature data. Point a) was attacked considering two reviewers that performed eligibility assessment independently and disagreements between those were resolved by consensus; all participants confirmed the process for data acquisitions. For point b), we examined results from the available studies for clues that suggest there may be missing studies (namely publication bias) or missing data from the included studies (namely selective reporting bias).

### Statistical analysis

All *CDH1 *germline mutations were stored in a database, considering the respective geographical origins. Qualitative data in the 2 groups were expressed as frequencies, organized into contingency tables, and evaluated with the [chi]^2 ^test (Pearson [chi]^2 ^test or Fisher exact test) to investigate potential differences. The results were considered statistically significant for *p *< 0.05. Statistical analysis was conducted by using SPSS statistical package (version 15.0) (SPSS, Chicago, IL).

## Results

### The majority of selected studies were original reports

We analysed a total of 212 studies; in detail 156 were excluded because they comprised somatic mutations, germline mutations in breast or colon cancers, molecular analyses, prophylactic gastrectomy descriptions and outdated reviews. Instead, the other 56 studies were included and classified as shown in the flow diagram (Figure 1). The majority of the studies were original reports (71.4%), and only the three (5.4%) latest updated reviews were considered for this study [4,5,54]. Two studies (3.6%) were the results of International Workshop Consensus Conferences (namely IGCLC), which delineated the clinical criteria selecting proband for the *CDH1 *genetic screening [2,3].

For each included study, we considered the number and the pattern of the identified *CDH1 *germline mutations; moreover, we carefully evaluated the ancestry of mutation carriers.

### Selection of countries with different risk area for gastric carcinoma

**Figure 2:**
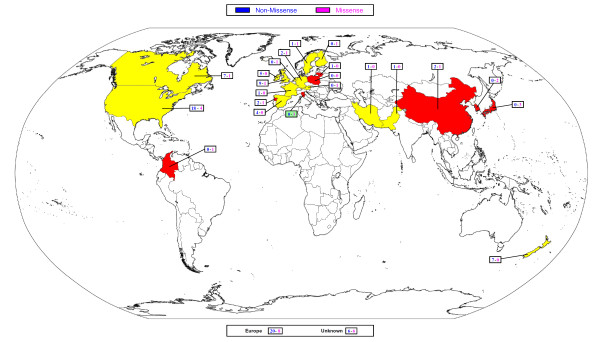
**Worldwide map with the overall *CDH1 *germilne mutations identified in the different countries**. Red and yellow indicate respectively middle/high- and low- risk area for gastric cancer development.

Far and Middle Western, European and American countries performed almost an independent genetic screening aiming for *CDH1 *germline mutation search (Figure 2). The countries that identified at least one germline *CDH1 *alteration were the following, a) Far East: China, Korea, Japan and New Zealand; b) Middle East: Iran and Pakistan; c) Europe: Italy, Spain, Ireland, the United Kingdom, Lithuania, Germany, Finland, the Netherlands, Sweden, Portugal and Austria; d) America: USA, Canada and Colombia.

We assembled these countries considering the incidence rates of gastric carcinoma spread and were classified as: a) low- (group A) and b) middle/high-risk (group B) area (details are described in method section).

### Frequency of CDH1 mutation is associated with specific gastric cancer risk area

**Figure 3:**
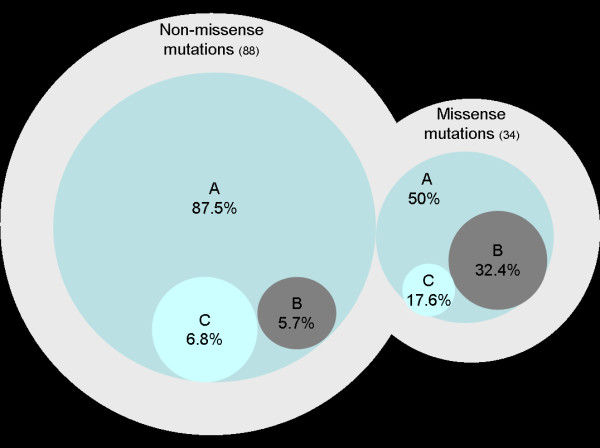
**The figure shows the distribution of missense and non-missense *CDH1 *germline mutations in low-risk area (group A) and high-risk area (group B)**. Ancestry information of group C is unknown.

All genetic screenings identified at least 122 *CDH1 *germline mutations (Table 1); 72.1% of these were non-missense and 27.9% missense alterations (Table 2). Non-missense mutations occurred with a frequency of 87.5% and 5.7%, respectively in groups A and B and missense alterations were 50% in group A and 32.4% in group B (Figure 3). Twelve *CDH1 *mutations, six non-missense and six missense, were reported without ancestry. 93 *CDH1 *germline mutations were identified in group A; 17 (18.3%) were missense and 77 (82.7%) non-missense. In group B, a total of 16 germline mutations have been reported, from which 11 (68.7%) were missense and 5 (31.3%) non-missense. The overall *CDH1 *germline mutation frequency was significantly higher in group A as well as the rate of non-missense alterations (group A vs. group B: Fisher exact test χ^2 ^15,872; *p *< 0.001).

**Table 2:** Frequencies of missense and non-missense *CDH1 *germline mutations in group A and B; statistical analysis reveals a strong association between high *CDH1 *mutation frequency with low-risk area for gastric cancer

	Missense mutations (%)	Non-missense mutations (%)
**Low Incidence (group A)**	17 (50)	77 (87.5)
**High Incidence (group B)**	11 (32.4)	5 (5.7)
**Unknown**	6 (17.6)	6 (6.8)
**Total**	34 (27.9)	88 (72.1)

### Minimization of risk bias

Point a) Two independent reviewers confirmed the results crosschecking all data, as well as the number and the ethnicity of *CDH1 *germline mutations; results were routinely discussed in scientific meetings and seminars. To facilitate the common consultation, all identified *CDH1 *alterations were recorded in Table 1. Point b) The heterogeneity of publications could be a risk bias; we considered only original reports that were the large majority of the analysed paper (71.4%), and reviews as other papers were considered only to confirm the results from those original reports. In particular, the last review [5] and one original paper [18] reported some *CDH1 *germline mutations namely as "unpublished results", this could reduce the risk bias related with the "missing studies". Considering the "selective reporting bias" as the missing data from the included studies, we assessed that the *CDH1 *mutations overlapped with the latest review [5] and with the selected original papers.

## Discussion

### Incidence of gastric carcinoma

Until recently, gastric carcinoma was the second most common cancer worldwide, but now, with an estimated 934,000 new cases per year in 2002 (8.6% of new cases), it is in fourth place behind cancers of lung, breast and colon and rectum. It is the second most common cause of cancer death (700,000 deaths annually). Almost two-thirds of cases occur in developing countries and 42% in China alone. The geographic distribution of GC is characterized by wide international variations; high-risk areas include East Asia (China, Japan and Korea), Eastern Europe, and parts of Central and South America. Incidence rates are low (< 10 per 100,000 in men) in Southern Asia, North and East Africa, North America, Australia, New Zealand and Africa [17,55].

In Europe, GC represents the fifth most common cancer, after colon and rectum, breast, lung and prostate, with an incidence of 149,000 per year and mortality rate of 116,000. The highest European risk area for GC is the Eastern zone with an incidence of 70,000 per year (Belarus area). Portugal and Italy represent one of the most high-risk European areas for stomach cancer, in particular, respectively, with incidence in 2008 of 41,100 and in Italy with incidence of 33,400 per year [17]. In Italy, Tuscany and central regions present a GC mortality rate of 4,2/10,000 inhabitants (2002 ISTAT-*The Italian National Institute of Statistics*).

### E-cadherin and diffuse gastric cancer

The *CDH1 *gene maps to chromosome 16q22.1 and consists of 16 exons that encode a 120-kDa protein called E-cadherin, which is a member of the transmembrane glycoproteins family. E-cadherin is expressed on epithelial tissues and is responsible for calcium-dependent cell-cell adhesion. Functionally, it is critical for establishing and maintaining polarized and differentiated epithelia through intercellular adhesion complexes. Human E-cadherin is considered an invasion suppressor, and under-expression of E-cadherin is correlated with the infiltrative and metastatic ability of the tumor [6].

In 1998 in New Zealand, a country that represents a low GC risk-area, Guilford and Colleagues identified firstly in Maori kindred the first *CDH1 *germline truncating mutation in cases with family history for diffuse gastric carcinoma [1]. Subsequently, several international countries reported other *CDH1 *germline mutations in HDGC or in sporadic EODGC with different ethnicities [4,54].

The definition of the HDGC syndrome was established in 1999 by the IGCLC that also delineated the criteria for the *CDH1 *genetic screening, as follows: 1) two or more documented cases of diffuse gastric cancer in 1st/2nd degree relatives, with at least one diagnosed before the age of 50, or 2) three or more cases of documented diffuse gastric cancer in 1st/2nd degree relatives, independently of age of onset [2]. Subsequently, the last consensus conference of Cambridge in 2010 [3], modified the criteria as: a) two GC cases in family, one confirmed DGC < 50; b) three confirmed DGC cases in 1st or 2nd degree relatives independent of age; c) age < 40; Corso and Colleagues recently proposed the cut-off age of onset at ≤ 35 in sporadic EODGC [4]; d) personal or family history of DGC and lobular breast cancer, one < 50.

In this study, we reported a total of 122 *CDH1 *germline mutations, respectively 94 (77%) in group A (low-risk area) and 16 (13.1%) in group B (middle/high-risk area) (12-9.9% mutations were without ethnicity information).

Revising these 122 *CDH1 *mutations, we observed some main findings: 1) the overall mutation rate is lower in group B rather than in group A (13.1% vs. 77%; *p *< 0.001); 2) group A shows a higher frequency of non-missense mutations; moreover, *CDH1 *non-missense mutations are extremely lower in group B (5.7%), in which missense variants are predominant (32.4%) (Figure 2).

Considering the first point, we stated that *CDH1 *germline mutations in GC middle/high-risk area are rather rarely identified respecting to low-risk area; as reported, in middle/high-risk area the probability to perform a *CDH1 *genetic screening with negative result is high enough [10-12], in which maybe operates environmental factors.

### Summary of evidence: CDH1 mutations and geographic variability

The abovementioned clinical criteria represents an important approach in selecting probands for the *CDH1 *genetic screening. Currently, different modifications have been appointed at these criteria [2,4,32], evaluating clinical and molecular results along the international consensus conferences; so far, the risk area vs. incidence of gastric carcinoma has never been discussed in these scientific meetings. We stated that "GC risk area and incidence" can represent an important emerging factor that relates strongly to the prognosis and other important behaviors of GC patients; ethnicity of GC patients coming from low- or high-risk areas could be considered in these criteria in order to identify cases with high or low-risk mutation carriers in hereditary syndromes. Information about the ancestry, can improve the accuracy of proband selection for the *CDH1 *genetic screening; in particular, we can consider GC patients as high or low-risk mutation carriers respectively in low or high-risk GC areas whenever the abovementioned criteria is fulfilled. This data verifies that in high-risk areas, clinical criteria should be applied strictly to cases with a strong family history for GC in particular when it has identified very young members.

It is important to stress that geographical variability represents a novel risk factor for gastric carcinoma, clinico-pathological features and some molecular results can confirm it. These acknowledgments, like the GC risk-area, provide important information and can propose different approaches in clinical practice for GC prevention and treatment.

### Molecular features and pathogenicity of E-cadherin missense mutations

The second consideration of this study is that GC high-risk area shows a higher frequency of *CDH1 *missense mutations; identification of a *CDH1 *missense alteration also implicated its functional assessment, because *in vitro *and *in silico *analysis with study of control population demonstrates that this mutation can be a novel polymorphic variant without pathogenic impact. We also revised the frequencies of *CDH1 *germline missense mutations with a pathogenic role that was assessed in 21 *CDH1 *missense alterations [4,56]; 17 of these showed a deleterious impact and respectively 5 (29.4%) were coming from middle/high risk-areas and 12 (70.6%) from low risk-areas. The pathogenicity of the remaining 13 mutations is unknown. As reported, the probability of finding a novel pathogenic *CDH1 *missense mutation (including also non-missense mutations) is higher in GC low-risk areas. Very recently, we revised the role of the *CDH1 *germline mutation in pathogenesis of sporadic EODGC; a total of 19 mutations have been reported to date in this GC group but only six (2.3%) of them did in fact represent variants with a proven potentially deleterious effect. This predicted pathogenicity was based on the type of mutation (frameshift) or on the results obtained from in vitro functional analysis. Five of the six germline pathogenic *CDH1 *mutations were detected in EOGC patients from low or moderate incidence GC areas, whereas only one arose in a patient from Portugal, a high incidence GC area. Although *CDH1 *germline mutations have been searched for in EOGC patients from other areas where high incidence rates of GC are verified, namely in countries like China, Korea, Japan, and even Italy, no deleterious *CDH1 *germline variants have been identified [4].

These results demonstrated that functional assessment of *CDH1 *germline missense mutations is mandatory, in particular in GC high-risk cases that show a higher frequency of unpathogenic missense mutations.

### Limitations of the current study

Certainly, this study presents some limitations that should be discussed, also for further consideration. First, the *CDH1 *germline mutation frequency could be affected by research activity, as publication bias or access to medical care, considering that some *CDH1 *variants could be unpublished in MEDLINE; this factor probably is due to the lack of a *CDH1 *recorded mutation database. However, MEDLINE is the unique search engine in which we can access easily and freely; unfortunately we do not have access in confidential and in unpublished data. However, this bias should be very small, because we carefully collected all *CDH1 *mutations recorded in MEDLINE; we also discussed this point in the "result" section. The second point is that we do not know precisely in what proportion of cases *CDH1 *mutations are identified in low vs. middle/high risk areas, and if the mutation frequency is related to the number of screened subjects; moreover, the accuracy of this study could be affected by the human migrations. The overall *CDH1 *mutation number identified to date is rather low and we are not able to also include this factor (as ratio between number of mutations vs. number of screened kindred, migration of population, etc.) risking to dissipate the statistical value, in the excess of data stratification. In the specific, we were not able to assess the quantitative genetic analysis due to the lack of information about the impact of environmental factor in DGC development and about migratory behavior. Further, if applicable and also considering that the clinical criteria are rather limited, in a larger family collection, this data could be considered. The third limitation is that we cannot analyse intestinal GC and other risk factors, such as environmental agents (i.e. specific diet habits); however, the aim of this study is only to assess the frequency of *CDH1 *germline mutation in DGC, in which environmental factors are probably less important than in intestinal hystotype. Moreover, the *CDH1 *germline or somatic mutations have not reported in intestinal gastric carcinoma, and for this reason, it was excluded from this systematic review.

## Conclusion

GC patients coming from high-risk areas show a lower incidence of *CDH1 *germline mutations when compared with individuals coming from low-risk areas. The finding that *CDH1 *mutation frequencies are extremely different in high and low-risk GC zones confirms that gastric carcinoma presents various clinico-pathologic and molecular features. This data could also open new different approaches in the GC prevention test; before proposing a proband candidate for the *CDH1*, genetic screening, geographic variability and family history should be considered.

## Competing interests

The authors declare that they have no competing interests.

## Authors' contributions

GC did *CDH1 *mutation research in MEDLINE and drafted the manuscript. DM performed statistical analysis. VP collected the overall *CDH1 *mutation in a database. CV was responsible of the correct histological classification of GC. FR conceived and designed the study, analysed the results and helped to draft the manuscript. All authors read and approved the final manuscript.

## Pre-publication history

The pre-publication history for this paper can be accessed here:

http://www.biomedcentral.com/1471-2407/12/8/prepub

## References

[B1] Guilford P Hopkins J Harraway J McLeod M McLeod N Harawira P Taite H Scoular R Miller A Reeve AE E-cadherin germline mutations in familial gastric cancer Nature 1998 392 6674 402 405 10.1038/32918 9537325

[B2] Caldas C Carneiro F Lynch HT Yokota J Wiesner GL Powell SM Lewis FR Huntsman DG Pharoah PD Jankowski JA MacLeod P Vogelsang H Keller G Park KG Richards FM Maher ER Gayther SA Oliveira C Grehan N Wight D Seruca R Roviello F Ponder BA Jackson CE Familial gastric cancer: overview and guidelines for management J Med Genet 1999 36 12 873 880 10593993 PMC1734270

[B3] Fitzgerald RC Hardwick R Huntsman D Carneiro F Guilford P Blair V Chung DC Norton J Ragunath K Van Krieken JH Dwerryhouse S Caldas C International Gastric Cancer Linkage Consortium Hereditary diffuse gastric cancer: updated consensus guidelines for clinical management and directions for future research J Med Genet 2010 47 7 436 444 10.1136/jmg.2009.074237 20591882 PMC2991043

[B4] Corso G Pedrazzani C Pinheiro H Fernandes E Marrelli D Rinnovati A Pascale V Seruca R Oliveira C Roviello F E-cadherin genetic screening and clinico-pathologic characteristics of early onset gastric cancer Eur J Cancer 2011 47 4 631 639 10.1016/j.ejca.2010.10.011 21106365

[B5] Guilford P Humar B Blair V Hereditary diffuse gastric cancer: translation of CDH1 germline mutations into clinical practice Gastric Cancer 2010 13 1 1 10 10.1007/s10120-009-0531-x 20373070

[B6] Suriano G Oliveira MJ Huntsman D Mateus AR Ferreira P Casares F Oliveira C Carneiro F Machado JC Mareel M Seruca R E-cadherin germline missense mutations and cell phenotype: evidence for the independence of cell invasion on the motile capabilities of the cells Hum Mol Genet 2003 12 22 3007 3016 10.1093/hmg/ddg316 14500541

[B7] Marrelli D Pedrazzani C Corso G Neri A Di Martino M Pinto E Roviello F Different pathological features and prognosis in gastric cancer patients coming from high-risk and low-risk areas of Italy Ann Surg 2009 250 1 43 50 10.1097/SLA.0b013e3181ad6487 19561483

[B8] Palli D Russo A Decarli A Dietary patterns, nutrient intake and gastric cancer in a high-risk area of Italy Cancer Causes Control 2001 12 2 163 172 10.1023/A:1008970310963 11246845

[B9] González CA Jakszyn P Pera G Agudo A Bingham S Palli D Ferrari P Boeing H del Giudice G Plebani M Carneiro F Nesi G Berrino F Sacerdote C Tumino R Panico S Berglund G Simán H Nyrén O Hallmans G Martinez C Dorronsoro M Barricarte A Navarro C Quirós JR Allen N Key TJ Day NE Linseisen J Nagel G Bergmann MM Overvad K Jensen MK Tjonneland A Olsen A Bueno-de-Mesquita HB Ocke M Peeters PH Numans ME Clavel-Chapelon F Boutron-Ruault MC Trichopoulou A Psaltopoulou T Roukos D Lund E Hemon B Kaaks R Norat T Riboli E Meat intake and risk of stomach and esophageal adenocarcinoma within the European Prospective Investigation Into Cancer and Nutrition (EPIC) J Natl Cancer Inst 2006 98 5 345 354 10.1093/jnci/djj071 16507831

[B10] Zhu ZG Yu YY Zhang Y Ji J Zhang J Liu BY Chen XH Lu Y Jiang HS Bu L Hu LD Kong XY Germline mutational analysis of CDH1 and pathologic features in familial cancer syndrome with diffuse gastric cancer/breast cancer proband in a Chinese family Eur J Surg Oncol 2004 30 5 531 535 10.1016/j.ejso.2004.03.004 15135482

[B11] Jakubowska A Lawniczak M Wojnarska B Cybulski C Huzarski T Byrski T Tołoczko-Grabarek A Jaworska K Durda K Starzyńska T Lubiński J CDH1 gene mutations do not contribute in hereditary diffuse gastric cancer in Poland Fam Cancer 2010 9 4 605 608 10.1007/s10689-010-9381-2 20842455 PMC2980631

[B12] Song W He YL Zhang CH Cai SR Zhou XF Peng JJ Wang Z Yang DJ Zhan WH Associations of E-cadherin gene (CDH1) and hereditary gastric cancer in China Zhonghua Wai Ke Za Zhi 2009 47 16 1204 1208 19781162

[B13] Graziano F Ruzzo AM Bearzi I Testa E Lai V Magnani M Screening E-cadherin germline mutations in Italian patients with familial diffuse gastric cancer: an analysis in the District of Urbino, Region Marche, Central Italy Tumori 2003 89 3 255 258 12908778 10.1177/030089160308900304

[B14] Corso G Pedrazzani C Marrelli D Pinto E Roviello F Familial gastric cancer and Li-Fraumeni syndrome Eur J Cancer Care 2010 19 3 377 381 10.1111/j.1365-2354.2008.01066.x 19674071

[B15] Corso G Roviello F Paredes J Pedrazzani C Novais M Correia J Marrelli D Cirnes L Seruca R Oliveira C Suriano G Characterization of the P373L E-cadherin germline missense mutation and implication for clinical management Eur J Surg Oncol 2007 33 9 1061 1067 10.1016/j.ejso.2007.03.001 17434710

[B16] Liberati A Altman DG Tetzlaff J Mulrow C Gøtzsche PC Ioannidis JP Clarke M Devereaux PJ Kleijnen J Moher D The PRISMA statement for reporting systematic reviews and meta-analyses of studies that evaluate health care interventions: explanation and elaboration PlosMedicine 2009 6 7 e1000100 10.1371/journal.pmed.1000100 PMC2707010 19621070

[B17] Ferlay J Parkin DM Steliarova-Foucher E Estimates of cancer incidence and mortality in Europe in 2008 Eur J Cancer 2010 46 4 765 781 10.1016/j.ejca.2009.12.014 20116997

[B18] More H Humar B Weber W Ward R Christian A Lintott C Graziano F Ruzzo AM Acosta E Boman B Harlan M Ferreira P Seruca R Suriano G Guilford P Identification of seven novel germline mutations in the human E-cadherin (CDH1) gene Hum Mutat 2007 28 2 203 17221870 10.1002/humu.9473

[B19] Bacani JT Soares M Zwingerman R di Nicola N Senz J Riddell R Huntsman DG Gallinger S CDH1/E-cadherin germline mutations in early-onset gastric cancer JMed Genet 2006 43 11 867 872 10.1136/jmg.2006.043133 PMC2563190 16801346

[B20] Oliveira C Senz J Kaurah P Pinheiro H Sanges R Haegert A Corso G Schouten J Fitzgerald R Vogelsang H Keller G Dwerryhouse S Grimmer D Chin SF Yang HK Jackson CE Seruca R Roviello F Stupka E Caldas C Huntsman D Germline CDH1 deletions in hereditary diffuse gastric cancer families Hum Mol Genet 2009 18 9 1545 1555 10.1093/hmg/ddp046 19168852 PMC2667284

[B21] Suriano G Yew S Ferreira P Senz J Kaurah P Ford JM Longacre TA Norton JA Chun N Young S Oliveira MJ Macgillivray B Rao A Sears D Jackson CE Boyd J Yee C Deters C Pai GS Hammond LS McGivern BJ Medgyesy D Sartz D Arun B Oelschlager BK Upton MP Neufeld-Kaiser W Silva OE Donenberg TR Kooby DA Sharma S Jonsson BA Gronberg H Gallinger S Seruca R Lynch H Huntsman DG Characterization of a recurrent germ line mutation of the E-cadherin gene: implications for genetic testing and clinical management Clin Cancer Res 2005 11 15 5401 5409 10.1158/1078-0432.CCR-05-0247 16061854

[B22] Oliveira C Bordin MC Grehan N Huntsman D Suriano G Machado JC Kiviluoto T Aaltonen L Jackson CE Seruca R Caldas C Screening E-cadherin in gastric cancer families reveals germline mutations only in hereditary diffuse gastric cancer kindred Hum Mutat 2002 19 5 510 517 10.1002/humu.10068 11968083

[B23] Richards FM McKee SA Rajpar MH Cole TR Evans DG Jankowski JA McKeown C Sanders DS Maher ER Germline E-cadherin gene (CDH1) mutations predispose to familial gastric cancer and colorectal cancer Hum Mol Genet 1999 8 4 607 610 10.1093/hmg/8.4.607 10072428

[B24] Moran CJ Joyce M McAnena OJ CDH1 associated gastric cancer: a report of a family and review of the literature Eur J Surg Oncol 2005 31 3 259 264 10.1016/j.ejso.2004.12.010 15780560

[B25] Humar B Toro T Graziano F Müller H Dobbie Z Kwang-Yang H Eng C Hampel H Gilbert D Winship I Parry S Ward R Findlay M Christian A Tucker M Tucker K Merriman T Guilford P Novel germline CDH1 mutations in hereditary diffuse gastric cancer families Hum Mutat 2002 19 5 518 525 10.1002/humu.10067 11968084

[B26] Guilford PJ Hopkins JB Grady WM Markowitz SD Willis J Lynch H Rajput A Wiesner GL Lindor NM Burgart LJ Toro TT Lee D Limacher JM Shaw DW Findlay MP Reeve AE E-cadherin germline mutations define an inherited cancer syndrome dominated by diffuse gastric cancer Hum Mutat 1999 14 3 249 255 10.1002/(SICI)1098-1004(1999)14:3<249::AID-HUMU8>3.0.CO;2-9 10477433

[B27] Shinmura K Kohno T Takahashi M Sasaki A Ochiai A Guilford P Hunter A Reeve AE Sugimura H Yamaguchi N Yokota J Familial gastric cancer: clinicopathological characteristics, RER phenotype and germline p53 and E-cadherin mutations Carcinogenesis 1999 20 6 1127 1131 10.1093/carcin/20.6.1127 10357799

[B28] Gayther SA Gorringe KL Ramus SJ Huntsman D Roviello F Grehan N Machado JC Pinto E Seruca R Halling K MacLeod P Powell SM Jackson CE Ponder BA Caldas C Identification of germ-line E-cadherin mutations in gastric cancer families of European origin Cancer Res 1998 58 18 4086 4089 9751616

[B29] Dussaulx-Garin L Blayau M Pagenault M Le Berre-Heresbach N Raoul JL Campion JP David V Bretagne JF A new mutation of E-cadherin gene in familial gastric linitis plastica cancer with extra-digestive dissemination Eur J Gastroenterol Hepatol 2001 13 6 711 715 10.1097/00042737-200106000-00016 11434599

[B30] Keller G Vogelsang H Becker I Hutter J Ott K Candidus S Grundei T Becker KF Mueller J Siewert JR Höfler H Diffuse type gastric and lobular breast carcinoma in a familial gastric cancer patient with an E-cadherin germline mutation Am J Pathol 1999 155 2 337 342 10.1016/S0002-9440(10)65129-2 10433926 PMC1866861

[B31] Kaurah P MacMillan A Boyd N Senz J De Luca A Chun N Suriano G Zaor S Van Manen L Gilpin C Nikkel S Connolly-Wilson M Weissman S Rubinstein WS Sebold C Greenstein R Stroop J Yim D Panzini B McKinnon W Greenblatt M Wirtzfeld D Fontaine D Coit D Yoon S Chung D Lauwers G Pizzuti A Vaccaro C Redal MA Oliveira C Tischkowitz M Olschwang S Gallinger S Lynch H Green J Ford J Pharoah P Fernandez B Huntsman D Founder and recurrent CDH1 mutations in families with hereditary diffuse gastric cancer JAMA 2007 297 21 2360 2372 10.1001/jama.297.21.2360 17545690

[B32] Brooks-Wilson AR Kaurah P Suriano G Leach S Senz J Grehan N Butterfield YS Jeyes J Schinas J Bacani J Kelsey M Ferreira P MacGillivray B MacLeod P Micek M Ford J Foulkes W Australie K Greenberg C LaPointe M Gilpin C Nikkel S Gilchrist D Hughes R Jackson CE Monaghan KG Oliveira MJ Seruca R Gallinger S Caldas C Huntsman D Germline E-cadherin mutations in hereditary diffuse gastric cancer: assessment of 42 new families and review of genetic screening criteria J Med Genet 2004 41 7 508 517 10.1136/jmg.2004.018275 15235021 PMC1735838

[B33] Avizienyte E Launonen V Salovaara R Kiviluoto T Aaltonen LA E-cadherin is not frequently mutated in hereditary gastric cancer J Med Genet 2001 38 1 49 52 10.1136/jmg.38.1.49 11332401 PMC1734712

[B34] Frebourg T Oliveira C Hochain P Karam R Manouvrier S Graziadio C Vekemans M Hartmann A Baert-Desurmont S Alexandre C Lejeune Dumoulin S Marroni C Martin C Castedo S Lovett M Winston J Machado JC Attié T Jabs EW Cai J Pellerin P Triboulet JP Scotte M Le Pessot F Hedouin A Carneiro F Blayau M Seruca R Cleft lip/palate and CDH1/E-cadherin mutations in families with hereditary diffuse gastric cancer J Med Genet 2006 43 2 138 142 15831593 10.1136/jmg.2005.031385 PMC2564630

[B35] Mateus AR Simões-Correia J Figueiredo J Heindl S Alves CC Suriano G Luber B Seruca R E-cadherin mutations and cell motility: a genotype-phenotype correlation Exp Cell Res 2009 315 8 1393 1402 10.1016/j.yexcr.2009.02.020 19268661

[B36] Yoon KA Ku JL Yang HK Kim WH Park SY Park JG Germline mutations of E-cadherin gene in Korean familial gastric cancer patients J Hum Genet 1999 44 8 177 180 10319582 10.1007/s100380050137

[B37] Rogers WM Dobo E Norton JA Van Dam J Jeffrey RB Huntsman DG Kingham K Chun N Ford JM Longacre TA Risk-reducing total gastrectomy for germline mutations in E-cadherin (CDH1): pathologic findings with clinical implications Am J Surg Pathol 2008 32 799 809 10.1097/PAS.0b013e31815e7f1a 18391748

[B38] Jonsson BA Bergh A Stattin P Emmanuelsson M Grönberg H Germline mutations in E-cadherin do not explain association of hereditary prostate cancer, gastric cancer and breast cancer Int J Cancer 2002 98 6 838 843 10.1002/ijc.10258 11948460

[B39] Zhang Y Liu X Fan Y Ding J Xu A Zhou X Hu X Zhu M Zhang X Li S Wu J Cao H Li J Wang Y Germline mutations and polymorphic variants in MMR, E-cadherin and MYH genes associated with familial gastric cancer in Jiangsu of China Int J Cancer 2006 119 11 2592 2596 10.1002/ijc.22206 16929514

[B40] Roviello F Corso G Pedrazzani C Marrelli D De Falco G Berardi A Garosi L Suriano G Vindigni C De Stefano A Leoncini L Seruca R Pinto E Hereditary diffuse gastric cancer and E-cadherin: description of the first germline mutation in an Italian family Eur J Surg Oncol 2007 33 4 448 451 10.1016/j.ejso.2006.10.028 17126523

[B41] Oliveira C Ferreira P Nabais S Campos L Ferreira A Cirnes L Alves CC Veiga I Fragoso M Regateiro F Dias LM Moreira H Suriano G Machado JC Lopes C Castedo S Carneiro F Seruca R E-Cadherin (CDH1) and p53 rather than SMAD4 and Caspase-10 germline mutations contribute to genetic predisposition in Portuguese gastric cancer patients Eur J Cancer 2004 40 12 1897 1903 10.1016/j.ejca.2004.04.027 15288293

[B42] Wilcox R Perpich M Noffsinger A Posner MC Cooper K Hereditary diffuse gastric cancer: multidisciplinary case report with review of the literature Pathol Res Int 2011 2011 1 5 10.4061/2011/845821 PMC3038690 21331337

[B43] Wang Y Song JP Ikeda M Shinmura K Yokota J Sugimura H Ile-Leu substitution (I415L) in germline E-cadherin gene (CDH1) in Japanese familial gastric cancer Jpn J Clin Oncol 2003 33 1 17 20 10.1093/jjco/hyg002 12604719

[B44] Mayrbaeurl B Keller G Schauer W Burgstaller S Czompo M Hoebling W Knoflach P Duba HC Hoefler H Thaler J Germline mutation of the E-cadherin gene in three sibling cases with advanced gastric cancer: clinical consequences for the other family members Eur J Gastroenterol Hepatol 2010 22 3 306 310 10.1097/MEG.0b013e32832bab9a 19474748

[B45] Barber M Murrell A Ito Y Maia AT Hyland S Oliveira C Save V Carneiro F Paterson AL Grehan N Dwerryhouse S Lao-Sirieix P Caldas C Fitzgerald RC Mechanisms and sequelae of E-cadherin silencing in hereditary diffuse gastric cancer J Pathol 2008 16 3 295 306 10.1002/path.2426 18788075

[B46] Jiang Y Wan YL Wang ZJ Zhao B Zhu J Huang YT Germline E-cadherin gene mutation screening in familial gastric cancer kindreds Zhonghua Wai Ke Za Zhi 2004 42 15 914 917 15363252

[B47] Rodriguez-Sanjuan JC Fontalba A Mayorga M Bordin MC Hyland SJ Trugeda S Garcia RA Gomez-Fleitas M Fernandez F Caldas C Fernandez-Luna JL A novel mutation in the E-cadherin gene in the first family with hereditary diffuse gastric cancer reported in Spain Eur J Surg Oncol 2006 32 10 1110 1113 10.1016/j.ejso.2006.06.006 16870389

[B48] Keller G Vogelsang H Becker I Plaschke S Ott K Suriano G Mateus AR Seruca R Biedermann K Huntsman D Döring C Holinski-Feder E Neutzling A Siewert JR Höfler H Germline mutations of the E-cadherin (CDH1) and TP53 genes, rather than of RUNX3 and HPP1, contribute to genetic predisposition in German gastric cancer patients J Med Genet 2004 41 6 e89 10.1136/jmg.2003.015594 15173255 PMC1735803

[B49] Salahshor S Hou H Diep CB Loukola A Zhang H Liu T Chen J Iselius L Rubio C Lothe RA Aaltonen L Sun XF Lindmark G Lindblom A A germline E-cadherin mutation in a family with gastric and colon cancer Int J Mol Med 2001 8 4 439 443 11562785 10.3892/ijmm.8.4.439

[B50] Simões-Correia J Figueiredo J Oliveira C van Hengel J Seruca R van Roy F Suriano G Endoplasmic reticulum quality control: a new mechanism of E-cadherin regulation and its implication in cancer Hum Mol Genet 2008 17 22 3566 3576 10.1093/hmg/ddn249 18772194

[B51] Ghaffari SR Dastan J Rafati M Sabokbar T Novel human pathological mutations. Gene symbol: CDH1. Disease: gastric cancer Hum Genet 2009 125 3 337 19309801

[B52] Caron O Schielke A Svrcek M Fléjou JF Garzon J Olschwang S Sézeur A Usefulness of prophylactic gastrectomy in a novel large hereditary diffuse gastric cancer (HDGC) family Am J Gastroenterol 2008 103 8 2160 2161 18796125 10.1111/j.1572-0241.2008.01982_17.x

[B53] Yabuta T Shinmura K Tani M Yamaguchi S Yoshimura K Katai H Nakajima T Mochiki E Tsujinaka T Takami M Hirose K Yamaguchi A Takenoshita S Yokota J E-cadherin gene variants in gastric cancer families whose probands are diagnosed with diffuse gastric cancer Int J Cancer 2002 101 5 434 441002E 10.1002/ijc.10633 12216071

[B54] Pedrazzani C Corso G Marrelli D Roviello F E-cadherin and hereditary diffuse gastric cancer Surgery 2007 142 5 645 657 10.1016/j.surg.2007.06.006 17981184

[B55] Holcombe C Helicobacter pylori: the African enigma Gut 1992 33 429 431 10.1136/gut.33.4.429 1582581 PMC1374052

[B56] Suriano G Ferreira P Mateus AR Correia J Henriques L Seruca R Genetics of hereditary diffuse gastric cancer: progress and future challenges Future Oncol 2006 2 3 363 370 10.2217/14796694.2.3.363 16787116

